# Spirit of the Foreign Periodicals, &c.

**Published:** 1845-04

**Authors:** 


### Periscope;

OR,

### CIRCUMSPECTIVE REVIEW.'

"Ore trahit quodcunque potest, atque addit acervo."

### Spirit of the Foreign Periodicals.

### Disease regarded as a False or Spurious Organisation. By Dr. Jahn.

There is a good deal of German mysticism in the following remarks, which are thereby rendered rather hard to be understood in certain passages. Our readers will however find no difficulty in tracing out the leading features of the doctrine which the learned writer seeks to establish.—(*Rev.*)

Since the experiments of Langenbeck have become generally known, the most incredulous must surely have ceased to doubt that the propagation of (many) diseases takes place in the same manner as that of the simplest forms of animal life. The opinion of Stark, that the production,—apart from the Contagion—of diseases must be regarded as an original generation *(generatio æquivoca)*, has been much objected to; but it seems to have been forgotten that the formation of the Infusoria, Vorticellæ, Fungi, &c., as morbid productions, occurs without contagion, and that these formations may, in the strictest sense, be regarded as instances of a veritable spontaneous generation.

A disease may justly be regarded as a *false* or *spurious* organisation; inasmuch as, besides the normal and primitive vital type, there is then present in the system a new and foreign type, which arises either from a transformation of the organic substance, or from a production of a new and extrinsic formation. This morbid type is developed and maintained at the expense of the normal life. A vast number of arguments and illustrations might be adduced to shew that the *prima causa mali* in (many) diseases is really and truly of an organic or animalcular nature: let us briefly consider a few of them.

There are certain morbid productions which almost every one must regard as genuine false organisations. Of this nature are—

1. The veritable morbid formations of animal life: for example, Entozoary *anthelminths;* various *mites*, the products of disease; various *infusoria*, whether the product of disease, or of spontaneous development.

2. Different vegetable productions; such as the *muscardine* of the silk-worm; the contagious *confervæ*, discovered by Hannover in and upon the aquatic salamander; the *fungi* found by Schoenlein existing in the patches of *porrigo lupinosa*, and which Remack has shewn may be propagated by inoculation; the various capillary *fungiform* productions observed by Fuchs and others in several exanthematous eruptions, &c. It would be difficult to admit that either these vegetable productions or their granules had entered the animal economy from without, and were not generated within. In the case of plants themselves, we have numerous instances of morbid vegetable formations, belonging to various groups of the Cryptogamic family, namely, to the *dartres*. We may mention, as belonging to this class, the *uredo, uromices, pharmegdium, puccinia, accidium, chrysomixa*, &c. &c.

Among the diseases of Plants, we cannot fail to recognise some under the form of *fungi*, which are generally considered as such, and are admitted into all systems of botanical arrangement: in this respect they are of the highest importance, as bearing upon the doctrine of the organic nature of diseases. The opponents of the doctrine of Spontaneous Generation will probably start the objection that the *fungi*, mentioned above as the products of disease, are never developed spontaneously in the bodies of plants themselves; but, on the contrary, that they attach themselves and become developed in the same manner as the genuine parasitic plants that come from without. The observations however of *Meyer* and *Unger* on the production of the Cryptogamic formations and of the mites of the cereal grains are, we think, unrefutable, and wholly at variance with this idea.

3. Among the spontaneous animal and vegetable morbid productions, we must enumerate the *Psorosperms* described by Müller, and the corpuscles (discovered by the same naturalist) which constitute a peculiar disease of the swimming bladder of the *Gadus Callurias*. Both of these formations well deserve notice; on the one hand, as affording a strong argument in favour of Equivocal Generation, and on the other, as having evidently a peculiar organisation, and being organised existences endowed with an individual life, and yet of such a nature as to forbid our classifying them among either plants or animals.

4. In the false or spurious organised products, we meet with formations which, on the one hand, are developed in the affected organism after the manner of its normal organs, and are associated with it; and, on the other hand, do not belong, and are foreign or even hostile, to it, seeing that they destroy its organic matter like parasitic growths.

They are organised, and exhibit a structure like that of normal organisms; they possess a determinate organic configuration, which often very closely resembles the form of regular or normal agents; like the ultimate organic elements, they consist of masses which are generally of a rounded shape, and are surrounded with a proper envelope. * * * * * These points have a proper vital course, proper periods, and peculiar processes of nutrition and secretion. Like many beings low in the scale of organic life, they finish by a softening and a dissolution of the mass. Moreover, they almost all possess the faculty of self-reproduction; for, alike in the diseased and healthy organism, they can propagate (by contagion) by means of molecules, which detach themselves from the general mass, and which, being then deposited upon another point, become developed and give rise to an affection altogether similar to that from which they were derived. These molecules possess so independent a vitality that they are capable of resisting the assimilative power of the organism. It is for this reason that it is generally so difficult, and often quite impossible, to cure them; for they possess a force of reproduction much greater than that of the organism and its different parts, being not unfrequently capable of reproducing themselves, even after they have been separated into invisible rudiments. They become fixed in the system, so that it is utterly impossible to trace any line of separation. This is clearly proved both by the morbid formations (*psorosperms* and others) discovered by Müller, by the uncertainty that still prevails as to the nature of Acephalocysts, and by the circumstance that certain naturalists have declared that several false organisations were entozoary productions.

5. The Exanthemata are closely allied to the spurious organic productions to which we have been alluding. Contagious diseases are, like false organisations, distinguished by their generative activity and their power of self-reproduction, by their fixed periods and terms of duration, and by the co-existence of other material productions that usually accompany them.

As the entire organism is composed of primitive molecules (cellules), and as it is a universal physiological law that certain material or organic changes attend every dynamic derangement, we must attribute all morbid alterations in the body to changes in the condition of these primitive formations. It has been shewn that in inflammation, typhus fever, chlorosis, scrofulous disease, scurvy and many other maladies, the globules of the blood undergo a decided change; that all sorts of external influences modify the form and colour of these globules; that in tabes dorsalis, the parts which compose the spinal marrow, and in atrophy, the molecules which compose the affected organ, are evidently more or less altered in their character; and that even in mental diseases we may often demonstrate a change in the conformation of the brain, manifested by an alteration of its primitive molecules. It is by the right application of the microscope and of chemical tests that we may hope to discover the determinate alterations in the primitive constituent elements of the organism. The production in diseases always takes place up to a certain point, independently of the idea of the individual organism; because the primitive molecules are not found in a tissue which occupies, or which has occupied, the morbidly affected point. Every disease therefore, whatever be its name, must be considered as consisting in a false or abnormal organic production.

The researches of *Langenbeck* on the contagious nature of carcinoma, and those of *Klenck* on that of tubercles, melanosis, condylomata, warts, coryza, carbuncle, hydrophobia, and the acute exanthemata, have clearly shewn that primitive, morbid and abnormal molecules may reproduce other like molecules; and, in short, that it is this very production of " molecules monstrueuses" which occurs in and constitutes the essential character of a contagious disease. The numerous examples of hereditary harelip, of supernumerary fingers and toes, and other vices of conformation, satisfactorily demonstrate that abnormal monstrous organised formations have the power of reproducing their like—a fact that quite accords with, and fully confirms, the accuracy of our position, that the morbid molecules in a living body are capable of producing molecules of a like nature to themselves.

As to the mode of development of those vegetable and animal *protorganisations*, which we have regarded in the preceding remarks as false organisations and morbid productions, our author suggests the following propositions to shew that they are formed from an altered or vitiated state of the organic molecules and primordial *cytoblastema.*

1. It has been observed by many naturalists that, in the lowest organised forms of the animal and vegetable world, there occur transformations from one nature into another. Now the primitive molecules of the organism are endowed with a proper and independent vitality in a high degree, so that they will sometimes continue to exist when transplanted into a different organism. This vitality, we may fairly presume, becomes still greater in certain favourable circumstances. Why then, seeing that we recognise a similar augmentation of the individuality of the primitive molecules, may not external influences advance a step further, and so act that the primitive molecules or their *cystoblastema* become transformed into beings that are completely individualised, into veritable protozoites et protophytes that are susceptible of a parasitic existence, as in the abovementioned transformations of the lowest forms of organised nature? There is produced in beings of a less perfect development the germ of other beings that are more perfect and complete. Now it is probable that a transmutation of the elementary molecules of the organism into beings possessing an individuality of existence may take place, as we have supposed. Various observations on certain animal and vegetable existences render this supposition highly probable.

2. The processes of fermentation and putrefaction are, according to the opinion of *Liebig*, movements of decomposition, in the former case of unazotised, and in the latter of azotised, substances. Both belong to what have been called chemical metamorphoses; *i. e.* to that class of phenomena in which an organic combination is decomposed by the chemical affinity of a second body, or by the influence of heat, or by some other cause, so that there are evolved from them two or more new compounds, and yet none of these elements is rendered free.

It is now ascertained that in fermenting fluids there become developed vegetable productions of an inferior order, and in putrescent animal matter certain infusoria, which, in the opinion of some distinguished naturalists, are generated by spontaneous formation; although, when once formed, they may propagate themselves by a process of vegetation. Now it is probable that not unfrequently those morbid conditions—which consist in the supervention of similar decompositions in some part of the body, in the *cystoblastema,* or in the primitive molecules of the fermentation and putrefaction—and consequently the chemical elements of the cystoblastema or of the molecules, experience a derangement in the attraction, which is indispensable to the continuance of the organisation in a healthy condition: this derangement being occasioned by certain external agencies which entirely overpower the dominion of the general vital powers. Among these derangements we may enumerate various diseases, gangrenescencies, putrescences, &c. But in these conditions there may possibly be developed—in consequence of the influence of external agencies, by spontaneous generation; in other words, in consequence of the ever-acting creative power of Nature on organic matter in a state of decomposition—certain powers or properties, in the same manner as in the processes of fermentation and putrefaction. On this, as on other points, it is to the morbid states of the vegetable world that we are to look for the most conclusive instances in the way of illustration.—*Haeser's Archiv. and Archives de la Medecine Belge, Juin,* 1844.

---

### On the Microscopic Texture of Cancer.

M. Desormeaux has recently published a valuable inaugural dissertation, entitled *Recherches sur la theorie elementaire de la production des tissus accidentels,* in which he has given an excellent summary of all the recent researches on the intimate structure of cancerous formations.

Müller, and (since the publication of his writings) most other pathologists, has arranged these morbid growths into two great families or groupes, viz. the Encephaloid and the Scirrhous. Of the former he makes the following three subdivisions.

1. Carcinoma medullare, *in which there is a predominance, in the medullary mass, of round globules over loose fibrous tissue.* The globules are of various sizes; but the smallest are larger than pus-corpuscles. Each contains a granular substance or nucleus within. They are very similar, in many respects, to those of common Cancer, and of reticulated Carcinoma or Scirrhus.

2. Carcinoma medullare, *consisting of pale, elliptic, non-elongated Corpuscles, and of a fundamental cerebriform mass.* These corpuscles are usually twice or three times as large as the globules of the blood. There is never any appearance of fibres proceeding from their surface, and they rarely exhibit any traces of nuclei within them.

3. Carcinoma medullare *with fibrated or fusiform corpuscles.* This species of Encephaloid structure has at times, on laceration, a sort of fibrous aspect, when the fusiform corpuscles are arranged in a somewhat determinate direction. According to the direction which they assume, the morbid mass will present a radiated or a tufted appearance. In many cases, indeed, their directions are so various that the lacerated surface exhibits no trace of fibres anywhere. The fusiform corpuscles are sometimes nucleated; at other times they contain granular points, but without distinct nuclei. They are elongated on one or two sides into fibres of different lengths. They may be considered as cells that are arrested at the period of the process of transition from the cellular to the fibrous condition.

The three forms of the disease now described may (most probably) be regarded as so many degrees or stages in the development of the same tissue; these successive stages being characterised, 1, by rounded nucleated globules; 2, by elongated oviform globules, which are either non-nucleated, or indistinctly so; and, 3, by fusiform globules.

These several kinds of globules may be regarded as so many successive epochs of evolution, through which a *cell* must pass before it can become a fibre. Thus we find that, in an Encephaloid mass, there is the same transformation of the primitive elements as occurs in many normal tissues—with this difference only, that the process of evolution is not complete, being arrested before the fibrine is perfectly formed. There is a perfect analogy in their mode of formation. The essential element of an encephaloid tumour is the presence of cells. In some cases the entire mass is composed of them, placed one alongside of the other, but without having any perceptible bond of union; while in others, there is a network of fibrous or cellular tissue interposed between the cells. When this fibrous tissue prevails, the Encephaloid then approaches in characters to the Scirrhous structure. In the latter, the existence of the two elements—cells and fibres—is always more distinctly marked than in the former. The fibres are often quite perceptible by the naked eye. Sometimes they are lengthened and run parallel to each other: at other times, they form rounded capsules, within which the globules are contained. As in the case of the newly-formed fibres of the cellular tissue, so those of a scirrhous formation are destroyed by acetic acid, leaving nuclei or nucleated fibres behind. The fibres sometimes exhibit at different points a sort of varicose enlargement, within each of which a nucleus is found. This appearance is often observed in fibrous tumours (not genuine scirrhus) of the uterus and other parts.

In the *reticular Carcinoma* of Müller, the white network, which encloses the scirrhous globules in its meshes, is formed of round opaque granulations, three or four times as large as the blood-globules; they are occasionally agglomerated into rounded masses. The genuine Scirrhous tissue, of a pale greyish colour, is composed of globules that, on the whole, resemble those of the first stage of an Encephaloid formation. These globules are either round or somewhat oval: along with them we find free *nuclei* with their *nucleoli.*—(Vogel.)

From a variety of observations we may reasonably conclude that the cells of Scirrhus are formed around the nuclei of which M. Vogel speaks; their contents are at first granular and almost opaque. When the process of softening commences, the granulations disappear, the globules become transparent, and within them are formed new cells, which at first are few in number, and gradually multiply, until they entirely fill the parent cell. M. Valentin, who, in part at least, admits this account of the progress of the cells, says, that the parent cells eventually burst and discharge their cellules: in this way we may account for the presence of young free cells in scirrhous formations that have become softened.

The intercellular substance seems to undergo certain modifications corresponding with the evolution of the cells; the granulations or granular points, which it often contains, usually disappear, and it becomes limpid, while at the same time the space, which it occupies, is diminished by the enlargement and multiplication of the cells.

The fibrous network does not appear to follow in its alterations the development of the cells: it may remain firm and resisting, while the cells are far advanced in their evolution. Even when a scirrhous tumour has become completely softened, this tissue sometimes forms shreds that retain their original character.

In *alveolar Cancer*, the basis of the morbid tissue consists of white fibres and lamellæ, which cross and intercross with each other, intercepting between the meshes thereby formed limpid cells, either closed or communicating with each other, of various sizes from that of a grain of sand to that of a large pea, and filled with a transparent gelatinous substance. In this substance there are cells, and these cells contain other cells more minute. The smallest of these cells exhibit at one point of their parietes a distinct dark-yellowish nucleus, and sometimes also many free and unattached granules floating within them. To this species M. Müller refers the *gelatiniform* and *areolar cancers* of Laennec and Cruveilhier. The cells of this species of the disease appear to be only an advanced or more mature degree of the cells of Scirrhus.—*Journal de Chirurgie de M. Malgaigne, Octobre,* 1844.

### Professor Berrutti on the Spontaneous Generation and Nature of the Spermatic Animalcules.

The reproduction of certain animal and vegetable species is effected, without the intervention of any ovum, after the manner of what is called gemmiparous and fissiparous generation. Now there is a great analogy between the development of *gemmæ* and that of *ova*. In the former, however, all the elements, that are necessary to the production of a living being, are found contained; whereas, the latter have need of the new elements of the prolific fluid before they can become duly evolved.

Every particle of a living being—which, on being detached from its parent body, is capable of reproducing an independent living creature—does not materially differ from a bud, either in its mode of origin, or in its property of producing new individuals. Thus organic molecules, in certain circumstances, have the power of attracting to themselves new materials from surrounding bodies, which they then incorporate with themselves, and so elaborate as to form a new being. Spontaneous generation, in the opinion of Professor Berrutti, consists in the exercise of this property, and differs from oviparous, gemmiparous and fissiparous generation, in that it takes place among molecules which, in consequence of the death of the parent, have ceased to constitute a part of a living individual.

Microscopic observations have clearly shewn that the globules, resulting from the dissolution of organic matter, possess an inherent activity: sometimes they approach to and unite with each other, and at other times they seem to be mutually repellent. It is not at all inconsistent with rational belief to suppose that this is the cause of spontaneous generation—an act, it may be observed, to which the concurrence of the atmospheric air and of water is probably always necessary.

The reproduction of parts of an organic body that have been excised or destroyed is effected by means of globules floating in a fluid, which subsequently evaporates, leaving the globules dry: hence the cellular production and origin of new tissues. The same process of assimilation is likewise that by means of which the fœtus is formed in the maternal ovum, as Wolf and Rolando have shewn.

The necessary condition, therefore, of every sort of generation, reproduction, and even of the nutrition of organised parts is invariably the presence of certain organic globules endowed with a plastic activity, and floating in a fluid exposed to the contact of the atmospheric air—which, in place of furnishing carbonic acid as it does to plants, supplies ammonia for the spontaneous generation of animals. The fluid in its turn supplies hydrogen and oxygen.

The organic productions of spontaneous generation are the most simple of all, because they spring from organic globules that are not expressly prepared for this purpose.

Professor Berrutti is of opinion that not only infusory, but also entozoary, animalcules are developed by spontaneous generation. He considers it too as highly probable that the *acarus scabiei* is the product, rather than the cause, of the itch. Lastly, he seeks to shew that the Zoosperms are not genuine animalcules, but rather organic molecules formed in the minute extremities of the spermatic tubes by the effect of an exuberant nutrition. The action of the Zoosperms seems to be very analogous to that of the Pollen in the fecundation of plants; and their movements may fairly be compared to those of this vegetable matter.—*Annali Univ. di Medicina, luglio,* 1844, *and Revue Medicale, Nov.* 1844.

---

### CERVICAL FISTULÆ, A REMNANT OF EMBRYOTIC ORGANISATION.

Dr. Munemayer of Verden has collected together the histories of fifteen cases of this congenital malady. The following are the conclusions which he has drawn from his researches on the subject.

1. Fistulæ of the neck always occur in the same locality; viz. in the anterior lateral part of the neck. Their orifice corresponds to the angle formed by the internal bundle of the sterno-mastoid muscle and the sternal end of the clavicle. It is rare that they are ever observed more within the edge of this muscle, or at all behind it.

2. Their orifice is always very small, and in some cases it is scarcely visible. The surrounding skin is usually more or less affected: sometimes it is red and projecting, while in other cases it exhibits longitudinal folds which become more prominent during the acts of respiration and deglutition.

3. The discharge is secreted by an internal membrane, the nature of which seems to be intermediate between serous and mucous tissues.

4. The opening of these fistulæ is always directed towards the œsophagus and pharynx. In some cases they make themselves a passage through these canals; while, in others, they terminate in a cul-de-sac: hence the division into such as are complete, and such as are incomplete. Their *trajet* is manifested externally by a sort of hardish cord that is readily perceptible by the finger, and is similar to the excretory ducts of the conglomerate glands of Wharton, Steno, &c. Those fistulæ, that are incomplete, are usually from half a line to two lines in depth.

5. Dr. Munemayer coincides in the opinion of Dr. Aycherson, that these cervical fistulæ are to be regarded as a remnant or vestige of the fœtal organisation; the parts, which represent the branchiæ in the embryo, being not completely closed. It is not always quite safe to close them up, especially when they are complete.—*Med. Wochenschrift, Juin* 1844, *and Revue Medicale, Dec.* 1844.

---

### M. FLOURENS ON THE DEVELOPMENT OF BONE.

The following three propositions embody the chief results of M. Flourens' recent investigations upon this most interesting subject of enquiry.

1. *Bone is formed by the periosteum.* 2. *It grows by the super-position of external layers.* 3. *The medullary canal is enlarged by the absorption of the internal layers of the bone.*

The experiments, on which the first of these propositions is based, were performed on dogs. A portion of one of the ribs was excised; removing only the bone, and leaving the periosteum behind. It was found that, after the expiry of a few days, a minute osseous nucleus was formed within the periosteum between the two divided ends of the rib. This nucleus became larger and larger, until at length it rejoined these ends, the one to the other, thus filling up the void space between them.

The numerous preparations, exhibited by M. Flourens at the Royal Academy, clearly shew that the new bone is formed in the periosteum; that, when it is first formed, it is completely insulated and apart from the old bone; and that it is only by its gradual development and extension that it ultimately reaches the two divided ends of the old bone, thus re-uniting them together.

The *second* proposition—*bone grows in size by the super-position of external layers*—was established by numerous experiments on dogs and rabbits. One of the tibiæ was exposed, the periosteum divided, and a ring of platinum wire was then passed around between the bone and its investing membrane. The wound being then closed and left undisturbed, it was found after a certain period that the new bone, that had been formed, fairly invested with its recently-deposited layers the platinum wire. The *third* proposition was equally satisfactorily made out by the preparations that were exhibited.—*Comptes Rendus—Encyclographie des Sciences Medicales, Oct.* 1844.

---

### On the Deposition of Carbonaceous Matter in the Tissue of the Lungs.

1. There is continually forming and accumulating in the lungs of man, during adult life, and more especially in old age, a certain amount of carbon in a state of the most minute subdivision.

2. This carbon, that exists even in the very substance of the pulmonary tissues, does not come from without.

3. Wherever it exists in sufficient quantity to form deposits of one millimetre in extent, the air-tubes, the blood-vessels, and the pulmonary tissues become transformed into a dark-coloured substance, which may occupy even more than one-half of the entire lungs.

4. The respiration no longer goes on in those parts which serve as a *matrix* to the carbonaceous deposit; there also, the phenomena of the circulation do not take place in the state of disease, and the process of inflammation is consequently never developed.

5. The successive accumulation of this carbon beyond a certain term is apt to cause death in old age, by rendering the pulmonary tissue more or less impermeable to the air.

6. The constant presence of this substance in the lungs of old persons is one cause of the fatality of pneumonia and congestive affections of the respiratory organs in them.

7. These molecules of carbon in the pulmonary parenchyma seem to have a marked influence on the phenomena, which may subsequently occur in and around tuberculous deposits. When tubercles are formed in the lungs, and the carbonaceous matter is deposited in considerable quantity around them, they do not undergo the successive changes proper to phthisis, in the usual course of this disease. The tubercles become calcareous, are deprived of their fatty matter, and do not enlarge. No vessels of new formation are developed around them; or rather, if such vessels have already become enlarged before the deposition of the molecular carbon, they become obliterated in consequence of this deposit, and the progress of the phthisical disease is arrested.

8. The production of carbonaceous matter in the lungs of man,—occurring, as it does, quite independently of any trade or profession and (most probably) of any particular sort of of food—is a fact which should be studied in a pathological as well as a physiological point of view, considering the influence which it may have on the course and issue of the most frequent pulmonic diseases to which old persons are liable. It would seem also that the deposition of this matter in the parenchyma of the lungs has a tendency to arrest the progress of phthisis, by forming a wall around the tubercles, and thus separating them from the intact pulmonary tissue.—*Comptes Rendus.—Revue Med. Dec.* 1844.

---

### ON THE TRANSPLANTATION OF THE CORNEA IN MAN.

M. Reisinger, in 1824, was the first who proposed to treat incurable central leucoma by substituting the clear cornea of a living animal for the (previously removed) cornea that was rendered opaque by disease. If we may quite credit his statement, he succeeded perfectly on one occasion (in a brute) by this method. Dieffenbach repeated the experiment several times; but in no case with success. Subsequently it has been tried by a good many different gentlemen, and with very various results. On some occasions the transplanted cornea has speedily mortified and fallen off, while in others it has more or less completely united, but shortly afterwards lost its transparency. In a few instances (it has been asserted) not only has this union taken place, but the transplanted cornea has remained perfectly clear.

M. Feldmann, in his recent critical examination of this question, is strongly inclined to doubt whether the operation has ever fairly succeeded; for, in all his experiments, the transplanted cornea has uniformly become not only opaque, but more or less irregular on its surface. It has only been within the last few years that an attempt has been made on the human subject to cure staphyloma in this way. Professor Wutzer of Bonn, and Dr. Kissam of New York seem to have performed the operation about the same time, and without either gentleman being aware of what the other was doing. The former made use of the cornea of a living sheep, to replace the diseased cornea that was excised. The transplantation, we are told, succeeded; but the cornea subsequently became quite opaque in consequence of the ophthalmia that ensued. The Professor repeated the operation on another patient, but with no better ultimate success.

The account of Dr. Kissam's case will be found in the New York Journal of Medicine for March, 1844. He made use of the cornea of a pig's eye, securing it in its new position with two ligatures placed in the direction of the palpebral commissures. For thirty-six hours after the operation, there was great chemosis of the eye-ball, so that it was difficult to ascertain the state of the parts. It was then found that the new cornea was adhering: the ligatures were therefore removed. The vision was improved immediately after the operation; but, as the humours of the eye were themselves unhealthy, it was very imperfect. The replaced cornea retained its transparency for a fortnight; then it became opaque, and ultimately it was absorbed.—*Journal de Chirurgie, Sept.* 1844.

N.B. There is an elaborate historical account of this operation in the *Archives Generales de Medecine* for May last.

---

### HYDROCELE OF THE NECK.

The tumours of the neck, which M. Maunoir has designated *Hydroceles,* and M. Percy *Hydrobronchoceles,* have often been confounded with genuine Goitre; and yet the two morbid states are very different, and they require very different modes of treatment.

As long as the tissue of the Thyroid gland is not decidedly altered, but is merely hypertrophied, we may reasonably expect to disperse the swelling by judicious treatment perseveringly employed. Not so, when any actual disorganisation has taken place.

The diagnosis is usually sufficiently easy. The experienced practitioner will not confound the hard, irregular, and lobulated masses, which are the result of the degeneration of the thyroid gland, with the smooth and uniform rounded tumours, unaccompanied with any change in the colour of the skin, and in which a fluctuation is more or less distinctly perceptible.

Sometimes, indeed, the gland, situated immediately in front of the neck, is *parsemée* with cysts which gradually dilate until they acquire an immense size; but even then it will be generally not difficult to distinguish their origin and real nature, and to discover in their circumference some parts of the organ in which they have become developed.

One cyst may attain such dimensions that it compresses the surrounding parts so much as even to cause their atrophy—a condition which probably existed in the second case which we propose to relate.

The parietes of the cyst present, in the majority of cases, so considerable a density or firmness that we are obliged to renounce the treatment by injecting a stimulating fluid into its sac; for this would only induce inflammation of the tumour, without being followed by any adhesion of the opposite surfaces, or obliteration of the cavity.

M. Maunoir was the first to recommend the use of the Seton; and most surgeons have followed his practice. In the following two cases, however, the knife was used instead; the success obtained fully justified the propriety of this mode of treatment.

*Case* 1.—A lady, 30 years of age, had for a length of time been distressed with sharp pain in the cervical region; her sleep was much disturbed, and her general health a good deal affected. Ever since the catamenia first appeared, there had been a fullness in the situation of the Thyroid gland. For many years it produced no inconvenience; but of late the patient was much incommoded by it. M. Fleury, when he first visited her, learned that, for some days previously, her breathing had become so much embarrassed that her attendants were afraid of suffocation. At this time the tumour of the neck was as large as the head of a full-grown child; it was quite smooth and uniform on the surface; the teguments were unaltered, except immediately in front, where they were attenuated and of a purplish hue, indicating incipient ulceration, and did not adhere to the subjacent mass, which appeared to be closely attached to the parts on which it rested. An indistinct feeling of fluctuation was perceptible on moving the tumour to and fro. On making an exploratory puncture, a few drops of a brownish-coloured serosity flowed out; the opening was enlarged with the bistoury, and a large quantity of this sort of fluid, holding in suspension a number of dark coagula, escaped. The patient experienced great relief from the removal of the pressure on the trachea and œsophagus.

Some time afterwards the following operation was performed, with the view of effecting a radical cure of the malady. Two semi-elliptic incisions were made so as to embrace the attenuated portion of the skin on the front of the neck; the integuments were then dissected back from the tumour on either side, and as much of the parietes of the cyst was excised as possible, with curved scissors; but its posterior wall adhered so firmly to the trachea that it could not be separated with safety. A very great number of bloodvessels required a ligature; many of them had become enlarged to a very considerable size. The wound was then filled with lint, and a roller was passed lightly round the neck. In the course of a few days, a healthy suppuration was established, and the internal surface of the wound began to be covered with granulations. A month after the date of the operation, the patient was able to leave her bed-room, and was quite free from the horrible pains from which she formerly suffered; all the inconvenience that remained was an unpleasant stiffness in moving the neck.

*Case* 2.—A young woman, 23 years of age, had, for 10 or 12 years before she consulted M. *Fleury*, been affected with sharp pains on the left side of the neck, which was considerably enlarged. Immediately in front of the trachea there was a tumour as large as a full-sized orange, and which, independently of the deformity thereby occasioned, distressed the breathing at intervals; and sometimes so much as to oblige her to remain in the upright position during the whole of the night: it appeared to adhere firmly to the air-tube. As I thought that I could feel a fluctuation, I proposed to practise an exploratory puncture. A long narrow bistoury was therefore introduced; and from the wound there issued a small quantity of a creamy-looking whitish fluid.

As little or no relief was obtained from this operation, I determined to adopt a similar line of procedure as in the former instance.

A vertical incision having been made along the entire length of the tumour, the cyst was freely opened, and gave vent to about a glassful of the same sort of fluid as had flowed out before. On introducing the finger, I felt a large sac, whose cavity extended upwards above the larynx and downwards to the sternum; its walls were very hard and resisting, and its inner surface was lined with a fibro-cartilaginous covering. This large pouch was filled with lint, after several small arteries were secured by ligature, and a gentle compression kept up by a roller passed round the neck.

A copious and rather fætid suppuration ensued, and continued for several days; but the character of the discharge gradually improved, and the state of the patient was altogether more satisfactory. Repeated injections of chloruretted water were found to be of great use. At the end of the fifth week, it was not necessary any more to introduce lint into the cavity; and all that was required was merely to leave a small portion of it in the orifice, in order to maintain this sufficiently open. Two weeks subsequently, the suppuration ceased entirely, and the wound healed.

M. *Fleury* considers these two cases as sufficient to shew that surgeons should not follow M. Maunoir in unreservedly condemning the treatment of Hydrocele of the neck either by simply incising the cyst and discharging its contents, or else by removing a certain portion of its parietes at the same time. It will be generally prudent to excise a small portion of the sac immediately in front; otherwise the wound will be apt to contract so much as to interrupt the free evacuation of the discharge.—*Annales de la Chirurgie.*

M. Bouchacourt has recently published two cases of encysted Goitre, or Hydrocele of the neck, of which he effected the cure first by puncturing and then (immediately afterwards) injecting a solution of Iodine—one part of the tincture to two, three, or four parts of water. In both cases, suppurative inflammation ensued, and the swelling required to be subsequently opened, in one instance with the knife, and in the other with caustic. In the latter case, the progress was slow, and for a length of time anything but satisfactory. Several months elapsed before the cure was complete. On the whole, we (Rev.) cannot regard the recorded experience of M. Bouchacourt as very encouraging as to the effects of the treatment which he recommends. He acknowledges that he was indebted to M. Velpeau for the suggestion of using the Iodine injection in such cases.—*Bulletin General de Therapeutique,* Sept. 1844.

### Great Malformation of the Heart; a single Auricle and Ventricle.

During the first six weeks of life, the child (the subject of the present case) seemed to thrive perfectly well; but then the breathing became difficult, and the surface of the face and body to exhibit a blueish hue. At six months, she was seized with convulsions, which were followed by Hemiplegia of the right side. This, however, gradually became less and less, and eventually the young sufferer recovered so well, that she could walk about with ease, after the right *tendo Achillis* (which had become contracted) was divided by M. Scoutteten. The dyspnœa and cyanosis were always increased upon any exertion; the blue tint was more *prononcé* on the right side. In her sixth year the girl died of an attack of Bronchitis. *Dissection.*—The substance of the two ventricles of the heart was nearly of the same thickness throughout. The septum was almost entirely awanting, there being no trace of it except at the lower part. The orifice of the pulmonary artery was separated from that of the aorta only by a small spur, which formed the upper part of the circumference of the interventricular opening. There was only one auriculo-ventricular orifice, common to the two ventricles and two auricles. These last-named cavities were separated from each other by a thin septum, which did not reach as far as this orifice, and therefore was incomplete. The *foramen Botalii* also was so open as to admit the point of the little finger. Thus it was that a free communication existed not only between the cavity of the ventricles and that of the auricles, but also from one auricle to the other. The auriculo-ventricular orifice was provided with a large triangular valve, that was attached to the anterior three-fourths of its circumference, and fixed at its apex to *columnæ carneæ* on the posterior part of the ventricular parietes. A few *columnæ*, proceeding from both ventricles, were attached to the two lateral borders of the valve.

In this case therefore, although there were distinct vestiges of four cardiac cavities, we may fairly say that the heart was simple—*i. e.* consisting of one ventricle and one auricle—as it exists in Batrachian animals. The presence of a single auriculo-ventricular orifice can leave no doubt on this point.—*Gazette Medicale*, No. 7, 1845.

---

### French Opinions on the Treatment of Typhus.

"In the treatment of our fevers, we have daily occasion to witness the pernicious effects of an irrational practice. There are still many physicians amongst us who almost invariably have recourse to the use of bloodletting and other debilitant remedies. The effect of such a course is inevitably to render the convalescence exceedingly tedious and often very imperfect. Many simple cases are thus converted into troublesome and unmanageable ones, in consequence of the impaired energy of the vital forces that has been induced. When the occurrence of the symptoms of adynamic weakness forces such practitioners to discontinue the use of their lowering regimen, they generally resort to the use of blisters and the administration of tonic medicines.

Let us briefly review some of the features of the disease.

There is usually a more or less considerable derangement of the stomach and bowels at first. Fortunately we have a remedy, or rather a class of remedies, which is exactly suited for the relief of such symptoms—we allude to emeto-cathartics: they generally act almost magically in removing the symptoms of the first stage of such fevers. After due evacuations both upwards and downwards, not only does the pyrexia usually subside, but all the phenomena of congestion and local irritation, that may have been present, are more or less completely relieved. The same decided and prompt benefit cannot indeed be expected from the use of these remedies, if the fever has already existed two or three days before they have been exhibited, and if any delirium be present: but even then the relief is sometimes very notable; the headache, stupor, and general prostration not unfrequently ceasing, or, at all events, being very materially diminished, after the action of the vomiting has entirely ceased."—*Gazette Medicale, Aout,* 1844.

We cordially assent to the practical truth and importance of these therapeutic instructions respecting the use of Emetics and Purgatives in the first stage of most typhoid fevers. We have often expressed our own opinions on this subject in the pages of this Journal, and strenuously urged our readers to recur to the practice recommended by the older physicians, and most religiously to eschew the evil ways of such advisers as the disciples of the Broussain school. Still, we are not inclined to go quite so far as our French brother, when he goes on to advise the administration (however guarded) of emeto-cathartic medicines in almost all stages of the fever of which he is treating. The paragraph, to which we object, runs thus:—"At a more advanced period of the disease, when the patients have fallen into an adynamic state, we must be more reserved in the exhibition of these remedies; not that they are not absolutely required or allowable; but only because the state of extreme debility usually present demands that the patient's strength be somewhat revived before they be given. Such cases require the use of stimulants—such as blisters, sinapisms to the abdomen and extremities—before we have recourse to the *heroic remedy*. However, as soon as the patient's strength is recovered, we should not delay longer, but resort at once to the exhibition of an emeto-cathartic: it is the sure means of preventing a fatal tendency." This seems to us dangerous advice. It is very rarely judicious to administer emetics in the advanced stage of febrile affections, when there is considerable prostration of strength; unless, indeed, the symptoms of gastric derangement are very obvious, and Nature herself makes an effort to get rid of the peccant secretions of the stomach and duodenum by the way of vomiting. The present is one out of many instances that might be adduced to shew how liable the French practitioners are to carry everything to extremes. A few years ago, the mere mention of an emetic in Typhus fever—which at that time was of a necessity a *gastro-enterite*—would have been denounced by nine-tenths of the physicians in Paris as incendiary and most dangerous practice. Now-a-days there seems to be a tendency to run to the other end of the race-course; and we should not be at all surprised to hear of ipecacuanha and tartar-emetic taking the heroic seat which has been so long occupied by leeches and "boissons adoucissantes."—*Rev.*

---

### PROFESSOR CORNEALINI ON THE PROXIMATE CAUSE AND TREATMENT OF CHLOROSIS.

From an extensive experience in the wards of the Pavia Hospital—to which he is attached as the professor of clinical medicine—the author deduces the following conclusions respecting this not unfrequent disease.

1. The essential nature of chlorosis consists of two pathological conditions, both of them appertaining to the solids:—the *first* being an inordinate excitation of the heart and arteries, and the *second* a chemico-vital alteration of the assimilative functions of Chylification and Hematosis. It is not possible to determine which of these two conditions is the primary and causal one.

2. No plan of treatment is so certain and efficacious as the exhibition of steel in some form or another: the preparations of this metal acting curatively upon both of the pathological states now mentioned.

3. There is no very marked difference in the comparative efficacy of different chalybeate preparations, except in so far as relates to their solubility in the animal fluids, and perhaps also to their readiness to become disaggregated by the process of digestion.

4. The addition of an acid decidedly increases the efficacy of steel remedies.

5. Steel-filings become converted, in the stomach of chlorotic patients, into the lactate of iron.

6. It is useless—and not unfrequently it is unsafe—to administer very large doses of ferruginous preparations.

Professor Corneliani has examined with great care the state of the blood in chlorosis; and most of his observations go to confirm the accuracy of MM. Andral and Gavarret's statements respecting the diminution of the normal proportion of red globules and hematosine.—*Annali Universali.*

---

### The Actual Cautery successfully Employed in Gangrene of the Mouth.

The case occurred in a female child, five years of age, during the convalescence from an attack of Typhus fever. The peculiar (by some deemed pathognomonic) offensive odour, that accompanies this species of gangrene, was remarked before any actual appearance of the disease was discoverable. There were four gangrenous patches on the upper and lower gums, besides one on the inner surface of the right cheek. Dr. Weber determined to apply the actual cautery to all the diseased spots, and the immediately adjacent parts. This was done, not without difficulty, as may be imagined; and the mouth was directed to be afterwards washed out every two hours with a decoction of Cinchona, to which some *spiritus cochlearea* was added. The offensive smell ceased immediately after the application of the cautery, and did not again return.

On the third day, the sloughy parts began to detach themselves; at the same time, a few suspicious-looking spots were touched with the nitrate of silver. Several teeth remained "*dechaussées,*" and at three points the maxillary (upper) bone was exposed. These necrosed portions, inclosing a molar tooth, and two smaller bits of the lower jaw were subsequently detached. During the progress of the case, an offensive purulent discharge took place from the ears, and numerous small abscesses formed on different parts of the body.

Dr. Weber does not think that, in this case (nor indeed generally), there was any reason to suspect that the gangrenous ulceration of the mouth was in any degree attributable to the action of mercury upon the system. He points out the analogy between this disease—which he designates by the name of *noma* or corroding ulcer—and the *pustule maligne,* so accurately described by Boyer and other continental writers.—*Gazette Medicale de Strasbourg,* Sept. 1844.

---

### M. Cazenave on the Different Sorts of Caustics.

The *Powder of Dupuytren* is composed of one part of arsenious acid and 200 parts of Calomel. It is a mild and very manageable caustic, that is useful in cases of Lupus in women and children, when the ulceration is superficial and of limited extent. If the diseased part be dry, it may be necessary to denude it by means of a blister, and then to sprinkle the powder upon the raw surface. A certain amount of heat and painful swelling is usually caused by this application. When it falls off, there is generally observed a decided modification of the diseased surface. A few applications are sufficient to effect a cure in a great many instances.

The *Vienna powder and paste* are remedies of great power in certain cases of lupous ulceration. They are composed of equal parts of powdered quicklime and potassa cum calce. In using it, we take a portion of this mixture, and add a small quantity of spirits of wine to bring the powder to the consistence of a paste. A piece of adhesive plaster, with a hole in it of the size of the intended eschar, should be laid over the diseased surface, and the paste is then applied upon the exposed part. It is to be left on for ten or twenty minutes, according to the depth of the eschar that is wished, and the ability of the patient to endure the pain.

The *chloruret of zinc paste* is much used in the present day. It is made by mixing one part of this substance with one or two parts of flour, moistening the mixture with as little water as possible. The pain produced by this application usually lasts for several hours. A greyish-coloured eschar is formed; and this, in most cases, remains attached for two or three weeks before it is separated. The surface underneath is generally not ulcerated. M. Cazenave very frequently has recourse to this caustic in certain cases of lupus, to destroy the non-ulcerated tubercles.

For this purpose, he usually applies only a very thin layer of the paste, so as not to destroy the entire tubercle; and in this manner he often succeeds in effecting a complete resolution of it, without any scar being left behind.

In very many cases of long-standing and deeply-corroding Lupous ulceration, he gives the preference to the Arsenical paste over the two others which we have mentioned: its action is two-fold; local as a caustic; and general, by becoming absorbed, and exercising a potent alterative or modifying influence upon the economy. The following is the formula which he invariably uses:—

Take of White oxyde of arsenic, 2 parts.
Sulphate of mercury, 1 part.
Animal charcoal in powder, 2 parts.

When used, a small quantity of this powder is to be made into a thin paste by the addition of a few drops of water; this is put upon the denuded surface—which should seldom or never exceed in extent that of a franc-piece. This application usually produces not only very sharp pain, but also a severe erysipelatous swelling, which lasts for 24 or 36 hours, and is sometimes accompanied with grave constitutional symptoms. Generally these subside very quickly; and then there remains on the cauterised part a hard brown crust, which often adheres for nearly a month, before it is detached.

*Fluid caustics.*—M. Cazenave frequently makes use of a solution of the Sulphate of Copper for the removal of those small warts that often form upon the shoulders and back, and also of certain pediculated horny productions, which occasionally appear upon these parts. A stronger solution must be used for the latter form of cuticular excrescence.

In the treatment of Favus and Tinea, he recommends a weak solution either of this salt of Copper, or of the nitrate of Silver, or of Acetic Acid.

Of fluid caustics, one of the most potent and useful is the acid nitrate of Mercury. When used to the surface pure and undiluted, it acts as a mere caustic; but when considerably weakened, and especially when applied to a large surface, it is unquestionably absorbed, and then it acts on the system.

It usually causes a good deal of pain and inflammatory swelling. The cases most benefited by its application are those of Lupus, in which the ulceration is extensive and not deep-seated.

The erysipelatous inflammation, which this as well as other caustics—more especially the arsenical paste—are apt to produce, need not be much dreaded; nay, the effects of the cutaneous Phlegmasia seem sometimes to be decidedly salutary in the end.—*Annales des Maladies de la Peau,* Oct. 1844.

M. Gibert has recorded in a recent No. (Oct. 1844) of the Revue Medicale, a case of severe scrofulous Lupus of the face, in which the progress of the disease was arrested and the extensive ulcerated surface became cicatrised under the employment, external as well as internal, of cod-liver oil. The use of this medicine was steadily persevered in for a full year and a half. During this time not only did the local malady become healed, but the general health—which had formerly been very weak and ailing—was very decidedly improved.

The patient was a young woman, and the disease had existed for nearly six years. On one occasion, she had derived very considerable benefit from the internal administration of the deuto-ioduret of Mercury, and the external use of the proto-ioduret ointment; but the benefit was temporary only. She had been subjected to a regular and protracted course of Iodine treatment; but certainly with no advantage.

---

### Corrosive Sublimate in Epilepsy.

In an elaborate article upon the Neuroses of the Ganglionic Nerves by M. Merat, Member of the Royal Academy of Medicine, we find the following remarks:—

"Since the year 1810, I have been in the habit of administering the Corrosive Sublimate in the treatment of several nervous diseases, and more especially of Epilepsy. I have witnessed decidedly successful results from its use in a good many—but certainly, it must be confessed, not in the majority—of the cases of this malady. The formula, which I use, will be found in the Dispensatory of Bouchardant (ed. 1840, p. 394). A pill, consisting of a sixteenth part of a grain of the Sublimate, one grain of Camphor, three-fourths of a grain of Opium, and half a grain of Musk, is given daily; this dose is to be increased every eight days by an additional pill of the same ingredients. Some patients have been able to take one or even two grains of the sublimate in the course of the twenty-four hours. As a general rule, I have never wished to exceed half a grain at most; unwilling to run the risk of inducing a black colour of the skin, as has happened to some patients after a protracted use of this drug.—*Revue Medicale,* Oct. 1844.

---

### Case of Dracunculus or Guinea-worm.

Towards the close of the year 1843, a man, who had returned from Senegal, was admitted into the St. Antoine Hospital, at Paris, for a small furuncular swelling on the dorsum of the left foot. It had existed for about a month, and was accompanied with intolerable itching in the part. An incision was made upon it, and next day M. Maisonneuve observed that a white filament projected from the wound: this was drawn out to the extent of about nine inches, and then it broke across. Another boil formed a little way below the head of the fibula; and as there was a tortuous, somewhat indurated, and painful subcutaneous line, extending from this point towards the calf of the leg, the skin was divided in two places, and an entire worm drawn out: it was of the thickness of a crow-quill, and upwards of two feet in length; it much resembled in appearance the *vas deferens.* The purulent matter from the two abscesses was carefully examined with the microscope; and, in that from the second one, there were observed myriads of minute animalcules, that moved about with great rapidity. On examining the worm itself, a milky fluid was perceived to be contained at one part of its tubular body.

In this fluid, too, numerous animalcules, similar to those which we have alluded to, were detected by the aid of the microscope. M. Maisonneuve very reasonably concludes that these embryotic Dracunculi may readily insinuate themselves under the skin in persons, who are in the habit of walking about with naked feet in such countries as that of Senegal. There is therefore no occasion to have recourse to the very questionable doctrine of Spontaneous Generation to account for the development of these subcutaneous entozoa. It is probable that the worm, when developed under the integuments, remains quiet until the period for discharging its ova has arrived, and then it makes an effort to perforate the skin, and become liberated for that purpose.—*Archives Generales de Medecine,* Dec. 1844.

---

### ON THE PERIODIC DISCHARGE OF OVA, AND THE FUNCTION OF MENSTRUATION.

The following propositions embody the most important conclusions that have been formed by the best authorities on this highly curious subject.

1. Menstruation commences at the period of the *maturity* of the ovules. 2. The final cessation of the catamenial secretion coincides with the abolition of the formative function of the germs. 3. The ovaries of women, who have ceased to menstruate, never contain the appearance of any vesicles that have recently burst, or that are about to do so (Negrier). 4. At each menstrual period, the highly excited state of the ovaries induces in the female a decided propension to coition. 5. The aptitude for fecundation is greatest on those days that immediately precede the menstrual discharge. 6. In all the lower animals, the ovaria become tumid during the season of rutting. 7. Women, in whom there is a congenital absence of the ovaria, never truly menstruate, however perfect may be the structure of the uterus and other parts of the generative system. 8. The extirpation of these organs puts a complete stop to menstruation, in casee where this function had been already established. 9. Women, in whom there is a congenital absence of the uterus, but in whom the ovaria are normally developed, experience every month all the phenomena of menstruation, the sanguineous discharge alone excepted. 10. The catamenial secretion ceases completely in women, in whom the ovaria have become affected with organic degeneration. 11. It has been asserted by some writers that lascivious girls have—in this respect like the common hen—occasionally discharged ova from the vagina, and that a mere voluptuous thought will suffice *pour ebranler* these minute vesicles. 12. In very many women, the menstrual period is preceded by severe colicy pains, attributable most likely to the turgid and excited state of the ovaries. 13. In those who suffer much at these periods, the cavity of the uterus sometimes becomes lined with a soft flocky membrane—a genuine *membrana caduca*—the formation of which is entirely independent of coition. 14. Lastly, in that singular case of monstrosity—in which the two girls, Helen and Judith, were united to each other by the posterior and lower parts of the back—the catamenial discharge took place in different quantities and at different times from each subject, although there was a complete anastomosis between the abdominal vessels of the two.—*Memoire pour servir à l'etude des Maladies des Ovaires, par Achille Chereau.* Paris, 1844.

---

### M. BISCHOFF ON THE SPONTANEOUS PERIODIC EXPULSION OF OVA.

This distinguished observer lays down the following law, which, it will be seen, brings the generative process in the mammiferous animals under the same general rule, which obtains in that of all other classes of animals.

"Both in mammalia and man also, the self-forming ova in the ovaries of the female experience a periodical ripening, entirely independent of the influence of the male semen. At this period, which, in animals, is called heat, and in women, usually, menstruation, these matured ova separate themselves from the ovary and are pushed out. At this time, alone in animals, and especially in women, are sexual desires also manifested. If coitus takes place, the fructification of the ovum follows, in consequence of the material action of the male semen upon it. If coitus does not take place, the ovum does not the less separate from the ovary, pass into the oviduct, but there perishes. The relations of time, however, as it appears, may vary in different animals within different yet fixed limits. The semen may have sufficient time to reach the ovary before the ovum has separated. The ovum may also have already passed out, and the semen have first encountered it in the oviduct; the action of the semen upon the ovum must still, however, always take place, if the ovum is to be developed, which development is commenced while it is still in the oviduct. But at this period of the periodical ripening of the ova, alone, can coitus be followed by fecundation."—Pp. 4, 5.

"During the years in which a woman is susceptible of impregnation, an ovum ripens and is separated from the ovary, every four weeks; this phenomenon being accompanied by simultaneous hæmorrhage from the uterus. This periodical maturation of an ovum is the first and most essential condition of conception and pregnancy. At this time alone will coitus be followed by conception; at all others this last will be impossible."—P. 43.

The assertion in the last paragraph is in accordance with the opinion of M. Pouchet—whose work, entitled "Theorie positive de la Fecundation, &c. Paris, 1843," we noticed some time ago.* "As it is more than sufficiently proved that in the mammifera it is at the epoch of sexual excitation that the ovules are expelled, and that at other times fecundation is impossible, it becomes equally evident, that, as the menstrual discharge in women represents this excitation, it is only in the neighbourhood of that period that our species possess the faculty of reproduction."

It has been generally believed that women, who do not menstruate, are incapable of conception. This opinion is, in all probability, strictly speaking, quite correct; but then it is to be remembered that the act of menstruation, or the monthly maturation and expulsion of an ovarian vesicle, may take place without being necessarily accompanied with a sanguineous discharge: the ovum may be emitted from the ovary without the slightest appearance of any outward hæmorrhage.—*The American Journal of the Medical Sciences*, Jan. 1845.

---

### Hygiene of Girls at the Period of Puberty.

"Under no circumstances should we seek to provoke the menses in girls, who are in the enjoyment of good health, even though they may have passed the mean age at which these usually appear; and for the very best reason, because all our efforts would be fruitless, as nature has not provided for their appearance. The power of ill health and sickness to retard the occurrence of puberty being satisfactorily established, we should seek to relieve any disease which may exist at the approach of that period, as the means employed for that purpose would probably act as the best emmenagogue. In one case only would it be proper

---

* Vide Medico-Chirurgical Review for January, 1844, p. 223.

to seek to favour the menstrual discharge in young girls indisposed at the age of maturity; and that is, when there is every reason to believe, from the present phenomena, that the Graafian vesicles are mature, but where the congestion of the genital organs, which then takes place, cannot relieve itself by the hæmorrhage—(p. 218.) To diminish this general or local plethora, which is one of the causes of this condition, and to give a direction of the blood towards the external genital organs, general bleeding and the application of leeches to the inner part of the thighs or vulva, with warm foot-baths, enemata, cooling regimen, &c., are advisable. The same means may be pursued to a great extent, where the retardation is consequent upon extreme excitement of the utero-ovarian nervous system. In these cases, which are attended with dragging pains in the groins and loins, and by engorgement and swelling of the uterus, the author recommends, with many previous writers, the use of ergot."—*American Journal.*

---

### ON CERTAIN DIFFERENCES IN THE COMPOSITION OF THE BLOOD IN THE MALE AND FEMALE.

MM. Becquerel and Rodier read a very elaborate memoir "on the Composition of the Blood in Health and in Disease," before the Royal Academy of Sciences, in the course of last November. As it must always be of the first importance to determine the normal condition of any of the fluids of the body, before we attempt to ascertain its morbid alterations, their remarks on the relative constitution of the blood in healthy adults of the two sexes may be deemed acceptable. The proportions given in the following table were determined by taking the average or medium figures obtained in a variety of experiments.

| | Man. | Woman. |
|---|---|---|
| Water .. .. .. . .. .. .. | 779 | 791,1 |
| Globules .. .. .. .. .. .. .. .. | 141,1 | 127,2 |
| Albumen.. .. .. .. .. .. .. .. | 69,4 | 70,5 |
| Fibrine .. .. .. .. .. .. .. .. | 2,2 | 2,2 |
| Extractive matters and free salts .. .. | 6,8 | 7,4 |
| Fatty matters .. .. .. .. .. .. .. | 1,6 | 1,620 |
| Seroline .. .. .. .. .. .. .. .. | 0,020 | 0,020 |
| Phosphorated fatty matter . .. .. | 0,488 | 0,464 |
| Cholesterine .. .. .. .. .. .. . | 0,088 | 0,090 |
| Soap .. .. .. .. .. .. .. .. | 0,004 | 0,046 |
| In 1000 parts of calcined blood. | | |
| Chloride of sodium.. .. .. .. .. .. | 3,1 | 3,9 |
| Soluble salts .. .. .. .. .. .. .. | 2,5 | 2,9 |
| Phosphates .. .. .. .. .. .. .. | 0,334 | 0,354 |
| Iron.. .. . .. .. .. .. .. | 0,565 | 0,541 |
| Density of the defibrinated blood.. .. | 1060,2 | 1057,5 |
| of the serum .. .. .. .. | 1028 | 1027,4 |

By comparing the two columns in this table, we find that certain very noticeable differences exist between the blood of the male and that of the female, in a state of health. The density of the defibrinated fluid is greater in the former, and consequently contains a larger quantity of soluble matters: the proportion of water too is decidedly less. The quantity of the red globules is considerably greater in the blood of the male than in that of the female: this is perhaps the most important, and indeed it is the fundamental, difference in the blood of the two sexes. In the female, the *minimum* number was 113, the *maximum* was 137, and the *medium* 127; (?) whereas in the case of the male, the *minimum* was 131, the *maximum* 151, and the *medium* 141.* The proportion of the Fibrine, and also that of the Albumen, was found to be very nearly the same in both sexes. The proportion of the iron present in the blood is always commensurate with that of the red globules.

MM. Becquerel and Rodier are of opinion that the function of *menstruation* exercises a marked influence on the proportion of the red globules in the blood of the female. In the girl, before this function has properly commenced, the relative quantity is below the normal standard; when the secretion is fairly established, it (the quantity) rises up to 127 or even higher; and this state of things continues until about the critical period of life, when menstruation ceases: then the proportion of the red globules falls considerably below this mark.

*Pregnancy* also has a very decided influence on the condition of the blood; the red globules and the albumen become diminished, and the fibrine, phosphorated fatty matter, and the water slightly increased.—*Encyclographie des Sciences Medicales,* Dec. 1844.

---

* This proportion is considerably higher than that (viz. 127) assumed by Andral and other hematological enquirers, as the standard of health; while the proportion of the fibrine in our table is lower than that (3) in theirs.

